# Can the Modified Frailty Index (mFI) Predict Intraoperative and Postoperative Complications in Older Women with Endometrial Cancer Undergoing Laparoscopic or Robotic Surgery? A Multicenter Observational Study

**DOI:** 10.3390/jcm12237205

**Published:** 2023-11-21

**Authors:** Chiara Schipa, Matteo Ripa, Valerio Gallotta, Andrea Russo, Lorenzo Polidori, Francesco Fanfani, Filippo Maria Capomacchia, Giacomo Corrado, Enrico Vizza, Anna Myriam Perrone, Liliana Mereu, Vito Cela, Francesco Legge, Georgios Hilaris, Tina Pasciuto, Marco D’Indinosante, Eleonora La Fera, Camilla Certelli, Valentina Bruno, Stylianos Kogeorgos, Pierandrea De Iaco, Konstantinos Lathouras, Liliana Sollazzi, Giovanni Scambia, Paola Aceto

**Affiliations:** 1 Department of Emergency, Anesthesiological and Reanimation Sciences, Fondazione Policlinico Universitario A. Gemelli IRCCS, 00168 Rome, Italypaola.aceto@policlinicogemelli.it (P.A.); 2 Catholic University “Sacro Cuore”, 00168 Rome, Italyfilippom.capomacchia@gmail.com (F.M.C.);; 3 Department of Ophthalmology, William Harvey Hospital, East Kent Hospitals University NHS Foundation Trust, Ashford TN24 0LZ, UK; 4 Department of Translational Medicine and Surgery, Università Cattolica del Sacro Cuore, 00168 Rome, Italy; 5 Gynecologic Oncology Unit, Department of Woman, Child and Public Health, Fondazione Policlinico Universitario A. Gemelli IRCCS, 00168 Rome, Italy; 6 Unit of Gynecologic Oncology, Department of Experimental Clinical Oncology, IRCCS “Regina Elena” National Cancer Institute, 00144 Rome, Italy; 7 Division of Gynecologic Oncology, IRCCS Azienda Ospedaliero, Universitaria di Bologna, 40138 Bologna, Italy; 8 Division of Obstetrics and Gynecology, Department of General Surgery and Medical-Surgical Specialism, University of Catania, CHIRMED Policlinico G. Rodolico, 95123 Catania, Italy; 9 Division of Gynecology and Obstetrics, Department of Clinical and Experimental Medicine, University of Pisa, 56126 Pisa, Italy; 10 Gynecologic Oncology Unit, Department of Obstetrics and Gynecology, “F. Miulli” General Regional Hospital, Acquaviva delle Fonti, 70021 Bari, Italy; 11 2nd Department of Gynecologic Oncology, Hygeia Hospital, Marousi, 15123 Athens, Greece; 12 Department of Obstetrics and Gynecology, Division of Gynecologic Oncology, Stanford University Hospital, Stanford, CA 94305, USA; 13 Data Collection G-STeP Research Core Facility, Fondazione Policlinico Universitario A. Gemelli IRCCS, 00168 Roma, Italy; 14 Department of Gynaecological Oncology, IASO General Hospital, Marousi, 15123 Athens, Greece

**Keywords:** frailty, modified frailty index, postoperative complications, intraoperative complications, endometrial cancer

## Abstract

Background: This study aims to evaluate the strength of the association between frailty and intraoperative/postoperative complications in patients undergoing minimally invasive surgery (MIS) for endometrial cancer. Methods: In this retrospective observational multicenter cohort study, frailty was defined beforehand by a modified frailty index (mFI) score of ≥3. Multiple logistic regressions were performed to investigate possible preoperative predictors—including frailty, age, and body mass index—of intraoperative and early (within 30 days from surgery) or delayed (beyond 30 days from surgery) postoperative complications. Results: The study involved 577 women, of whom 6.9% (n = 40) were frail with an mFI ≥ 3, while 93.1% (n = 537) were non-frail with an mFI of 0–2. Frail women had a significantly higher rate of intraoperative complications (7.5% vs. 1.7%, *p* = 0.01), with odds 4.54 times greater (95% CI: 1.18–17.60, *p* = 0.028). There were no differences in the rate of early postoperative complications (15% vs. 6.9%, *p* = 0.06) and delayed postoperative complications (2.5% vs. 3.9%, *p* = 0.65) for frail versus non-frail patients. The odds of early postoperative complications increased by 0.7% (95% CI: 1.00–1.15) for every one-unit increase in age (*p* = 0.032). Conclusions: Frailty was associated with a significantly higher risk of intraoperative complications in older women undergoing MIS for endometrial cancer. Likewise, increasing age was an independent predictor of early postoperative complications. Our findings support the practice of assessing frailty before surgery to optimize perioperative management in this patient population.

## 1. Introduction

Nowadays, due to the increase in the elderly population, aging plays a crucial role in the increased incidence of adverse outcomes in most clinical situations, including surgical settings. Furthermore, despite being a physiological process, aging is related to many comorbidities, such as cerebrovascular and cardiovascular disorders, which may deteriorate the overall health status [1]. In addition, the aging process is linked to frailty, a geriatric syndrome characterized by a condition of reduced functional reserve with increased vulnerability to stressors and limited ability to maintain homeostasis. This condition makes surgical procedures burdensome, thus potentially resulting in life-threatening complications [2,3,4,5].

Nowadays, the number of geriatric gynecologic oncology patients is continuously increasing. Therefore, clinicians dealing with elderly frail patients need to consider frailty when making therapeutic decisions [6].

Many grading scores, such as the modified frailty score (mFI), have been developed as screening tools [4]. Moreover, the mFI has been shown to predict the likelihood of postoperative mortality and morbidity in several surgical subspecialties, including gynecology [7]. In addition, many studies have confirmed that the mFI may predict postoperative complications and hospital readmission [8,9,10]. 

Despite the widespread use of frailty score systems and a recent systematic review showing that the mFI appeared as the most feasible tool for tailoring therapeutic strategies in patients with three or more frailty-defining items, only a few publications have assessed the frailty score systems’ ability to predict outcomes in gynecologic oncology surgery [11,12,13,14].

In addition, the impact of frailty on surgical outcomes has never been evaluated in a homogeneous sample of women with endometrial cancer. Thus, identifying frail surgical patients is essential for implementing “individualized” prehabilitation programs to enhance the potential of radical surgery and improve both clinical and oncological outcomes [9].

Therefore, our study evaluated the strength of the association between frailty, calculated using the mFI, and intraoperative or postoperative complications in patients undergoing minimally invasive surgery (MIS) for endometrial cancer.

## 2. Materials and Methods

### 2.1. Study Design and Population

This is a retrospective observational multi-institutional cohort study involving data from seven institutes: Fondazione Policlinico Universitario A. Gemelli of Rome (Italy), Regina Elena National Cancer Institute of Rome (Italy), Santa Chiara Hospital of Trento (Italy), Azienda Ospedaliero-Universitaria di Bologna (Italy), University of Pisa (Italy), “Miulli hospital” of Acquaviva delle Fonti in Bari (Italy), and Hygeia Hospital, Marousi, Athens (Greece). 

Each participating center independently obtained approval for conducting the study according to local and international legislation (Declaration of Helsinki).

Data from patients aged 70 and older affected with endometrial cancer who underwent MIS between 1 April 2002 and 31 October 2018 were analyzed according to previously published studies reporting the comorbidity incidence relevant to the surgery [15,16].

Specifically, in the majority of selected centers, patients underwent either laparoscopic or robotic surgery (Da Vinci Si or Xi, Intuitive Surgical Sunnyvale, Sunnyvale, CA, USA), with the surgical strategy chosen based on the patient’s clinical conditions or the surgeons’ choice.

All patients underwent a comprehensive examination before surgery, including medical history and physical and vaginal–pelvic examination. In addition, multimodal imaging, such as chest X-ray, ultrasound scans, pelvic magnetic resonance imaging (MRI), or computed tomography (CT) scans, was performed according to clinical practice. 

The relevant comorbidities, data from the surgical procedure, and lymph node assessment (i.e., systematic lymphadenectomy, lymph node sampling, or the sentinel lymph node technique (SLN)) were recorded for each patient. The Common Terminology Criteria for Adverse Events (CTCAE) version 5 was used to define intraoperative and postoperative complications [17].

Following a multidisciplinary tumor board discussion, including specialists from different fields, the adjuvant therapy was adjusted according to the pathologic findings from the primary surgery.

The National Comprehensive Cancer Network’s (NCCN) recommendations and ESGO and ESTRO guidelines were used to guide treatment [18]. 

All patients’ data were collected using REDCap, hosted at Fondazione Policlinico Universitario A. Gemellli, IRCCS, Rome, Italy [19].

### 2.2. Intraoperative Anesthesiological Management

Following a thorough clinical evaluation, all patients received standard basic monitoring and the same anesthetic regimen (general anesthesia). 

More specifically, oxygen saturation, 5-lead electrocardiography, non-invasive/invasive pressure monitoring and neuromuscular assessment (NMT neuromuscular transmission mechanosensor, GE Healthcare, Hertfordshire, UK), Bispectral Index (BIS VistaTM, Aspect Medical System Inc., Norwood, MA, USA), and diuresis were evaluated to monitor the patients intraoperatively. 

Anesthesia was induced using propofol (2–3 mg/kg), fentanyl (2–3 mcg/kg), and rocuronium (0.6–1.2 mg/kg). Sevoflurane at BIS-guided concentration (40–60) and remifentanil (0.05–0.2 mcg/kg/min) were used for anesthesia maintenance.

Lung protective ventilation was performed, maintaining a tidal volume of 6–8 mL/kg, with a respiratory rate of 10–14 bpm and a positive end-expiratory pressure of 5 cmH_2_O.

In order to keep patients’ temperatures within the normal range, a forced-air warming system (Bair Hugger Model 505, Arizant Healthcare Inc., St. Paul, MN, USA) and a fluid-warming device (enFlowR, BD, Franklin Lakes, NJ, USA) were utilized.

At the end of the surgery, in order to achieve a train-of-four (TOF) ratio of 0.9, the following drugs were used for neuromuscular reversal: neostigmine (up to May 2013) or sugammadex (since June 2013) [20]. Finally, an elastomeric pump carrying tramadol (5 mg/mL; 2 mL/h) and ropivacaine 0.2% (0.2 mL/kg) for wound infiltration was employed for postoperative analgesia. No patients received neuraxial anesthesia (spinal/epidural) combined with general anesthesia for pain control. No adverse events related to anesthetic drugs were reported during the study period.

### 2.3. mFI-11

Frailty was determined for each patient utilizing the mFI-11, which foresees 11 variables as indicated in the Canadian Study of Health and Aging Frailty Index: “a history of diabetes mellitus, chronic obstructive pulmonary disease or pneumonia, percutaneous coronary intervention, stenting, or angina, congestive heart failure, myocardial infarction, hypertension requiring medication, peripheral vascular disease or ischemic rest pain, cerebrovascular accident with neurological deficits, impaired sensorium, transient ischemic attack or cerebrovascular accident and functional status of ≥2” [7]. Based on the previous literature, patients with an mFI score of ≥3 were considered frail [21].

### 2.4. Statistical Analysis

SPSS v.27 was used for all statistical analyses (IBM SPSS, Chicago, IL, USA). Patients were divided into two cohorts for data analysis: non-frail (mFI 0–2) and frail (mFI of ≥3). Absolute and percentage frequencies were used to express qualitative variables. To analyze the distribution of quantitative variables, the Shapiro–Wilk test was utilized; normally distributed data are described as the mean and standard deviation (SD). The Chi-square test was used to compare between-group differences for each parameter, and the Student’s *t*-test was used to compare quantitative variables. In addition, three multiple logistic regressions were performed to investigate possible preoperative predictors—including frailty (yes/no), age, and body mass index (BMI)—of intraoperative, early, and delayed postoperative complications, respectively. The model did not include variables involved in the mFI calculation (medical comorbidities) to avoid less-reliable statistical inferences, as they were highly correlated with mFI (multicollinearity). Odds ratios and 95% confidence intervals were calculated, and the level of statistical significance was set at *p* < 0.05.

## 3. Results

### 3.1. Patient Clinical Characteristics

Patient demographics are displayed in Table 1. The mean patients’ age was 76.45 ± 4.72 years, whereas the mean BMI was 29.39 ± 5.92. Out of 577 women, 377 (65.3%) patients had hypertension, 130 (22.5%) had diabetes mellitus, 39 (6.8%) had heart failure, and 28 (4.9%) presented sensory deficits. Regarding histology, the most common histotype was endometrioid (n = 491, 85.1%), while the rarest ones were neuroendocrine (n = 1, 0.2%) and adenosarcoma (n = 1, 0.2%). 

Out of 577 women, 93.1% (n = 537) had mFI scores ranging from 0 to 2 and were considered non-frail, while 6.9% (n = 40) were considered frail with mFI scores of ≥3. More specifically, the mFI scores were 0 in 23.1% (n = 133), 1 in 49.4% (n = 285), 2 in 20.6% (n = 119), 3 in 4.5% (n = 26), 4 in 1.6% (n = 9), 5 in 0.4% (n = 2), and 6, 7, and 9 in only one patient, respectively (Table 2). There were no significant differences in median age (76.44 ± 4.75 vs. 76.55 ± 4.27, *p* = 0.89) and BMI (29.35 ± 6.99 vs. 29.91 ± 4.93 *p* = 0.56) between the cohorts. Analyses within both frail and non-frail groups showed no significant statistical difference in histology and grading. Frail patients were more likely to have diabetes mellitus (62.5% vs. 19.6%, *p* < 0.001), stroke (12.5% vs. 0.2%, *p* < 0.001), coronary heart disease (42.5 vs. 0.2%, *p* < 0.001), and heart failure (50% vs. 3.5%, *p* < 0.001) compared to non-frail patients.

### 3.2. Perioperative Outcomes

**Figure 1:**
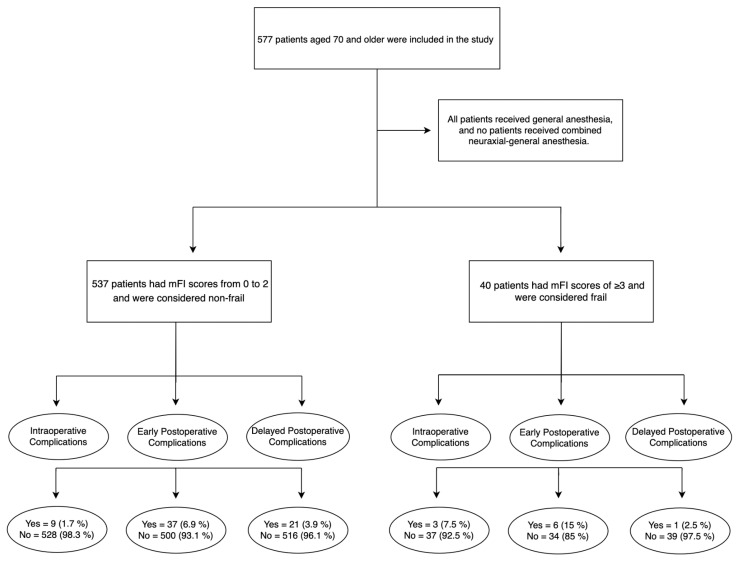
Flow chart.

The main results are shown in Figure 1. Frail women had a significantly higher rate of intraoperative complications than non-frail patients (7.5% vs. 1.7%, *p* < 0.01).

There was no difference in the rate of early postoperative complications (15% vs. 6.9%, *p* = 0.06) and delayed postoperative complications (2.5% vs. 3.9%, *p* = 0.65) for frail versus non-frail patients. All intraoperative complications were classified as grade < 3, according to the CTCAE.

There were twelve cases of intraoperative complications, including iliac artery injury (n = 1), vaginal laceration (n = 3), bowel injury (n = 3), bladder injury (n = 3), bleeding (n = 1), and hypertensive crisis (n = 1). In addition, three intraoperative complications (bowel injury n = 1, vaginal laceration n = 1, bleeding n = 1) were found in frail patients, and nine of them in non-frail patients (vaginal laceration n = 2, bowel injury n = 2, bladder injury n = 3, iliac artery injury n = 1, and hypertensive crisis n = 1).

Regarding early postoperative complications, there were five grade 3 complications (two bladder-vaginal fistulae, one bowel perforation, and two urinary site infections). 

According to the CTCAE, three late-postoperative complications were found (one bowel perforation and two incisional hernias). In addition, there were 10 laparotomic conversions due to obesity and excessive visceral adipose tissue (n = 5), vessel infiltration by the tumor (n = 2) or lesion (n = 2), and sigma infiltration (n = 1) (Table 3).

### 3.3. Multiple Logistic Regression Analyses

Regarding the intraoperative complications, the multiple logistic regression model explained 5.1% of the variance in intraoperative complications and correctly classified 97.8% of cases. Frail patients were 4.54 times more likely to exhibit intraoperative complications than non-frail ones (95% CI: 1.18–17.60, *p* = 0.028). 

The multiple logistic regression model was statistically significant for early postoperative complications, χ^2^(3) = 8.23, *p* < 0.0005. The model explained 3.5% of the variance in intraoperative complications and accurately identified 92.5% of cases.

Increasing age was associated with an increased likelihood of exhibiting early postoperative complications. In this regard, it was found that holding all other predictors constant, the odds of early postoperative complications increased by 0.7% (95% CI 1.00–1.15) for every one-unit increase in age (*p* = 0.032) (Table 4).

## 4. Discussion

This study showed that frailty, defined by an mFI score ≥ 3, was associated with a significantly higher incidence of intraoperative complications in 577 women undergoing MIS for endometrial cancer. Indeed, frail patients had a 4.54-fold increase in the odds of having intraoperative complications (95% CI: 1.18–17.60, *p* = 0.028). 

Previous studies, different from our analysis, demonstrated exclusively the predictive value of the mFI in assessing postoperative surgical outcomes [13,21]. 

Using the NSQIP database, Uppal and colleagues reported that the mFI predicted the critical-care support requirement and 1-month mortality in an analysis of 6551 women undergoing robotic surgery [21]. Furthermore, Chambers and colleagues recently demonstrated that the mFI was predictive of postoperative complications following cytoreductive surgery with hyperthermic intraperitoneal chemotherapy in patients with advanced or recurrent gynecologic cancer [13]. 

Patients with advanced ovarian cancer frequently exhibit frailty, which is independently linked to lower surgical outcomes and shorter overall survival [22,23]. Additionally, Mullen and colleagues showed, in a retrospective cohort of 163 obese women undergoing laparotomy via midline vertical incision, that frail patients had a significantly higher risk of wound complications, despite controlling for BMI, tobacco use, and perioperative glucose levels [24]. 

Although the mFI has been widely adopted to evaluate frailty severity in elderly women with endometrial cancer [14,25,26], different frailty assessment tools such as the FFC (Fried frailty criteria), Johns Hopkins Adjusted Clinical Groups (ACG) frailty-defining diagnosis indicator, and a combination of different global health assessment tools have been seldom adopted [27,28,29,30,31].

The FFC was used by Courtney-Brooks and colleagues to evaluate whether frailty may predict surgical complications among elderly women undergoing gynecologic oncology treatments. The rate of 30-day postoperative complications rose with the frailty score, reaching 67% for frail women [27].

In addition, in 2017, Driver and colleagues used a collection of health deficits as markers of frailty, such as albumin < 3.5 mg/dL, hemoglobin (Hb) < 10 mg/dL, body mass index (BMI) < 20 kg/mq, unintentional weight loss, Eastern Cooperative Oncology Group performance status (ECOG) ≥ 2, history of osteopenia or osteoporosis, and the Charlson comorbidity index, to evaluate frailty on a dichotomous scale (i.e., non-frail: no deficits; frail: at least one out of seven considered deficits). They found that frailty markers predict disease-free survival (DFS) and overall survival in elderly women with endometrial cancer [28].

In 2022, Nakhla and colleagues adopted the ACG frailty-defining diagnosis indicator that includes 10 clusters of frailty-defining diagnoses (malnutrition, dementia, impaired vision, decubitus ulcer, incontinence of urine, loss of weight, poverty, barriers to access of care, difficulty in walking, and falls). Their analysis showed that frailty independently predicted increased odds of respiratory, neurologic, renal, and infectious complications [29]. In addition, according to ACG frailty diagnosis indicators, Sia and colleagues showed that frailty was associated with an increased risk of intensive level of care, nonroutine discharge, and inpatient mortality during index admission [30]. 

Finally, in 2023, Anic and colleagues evaluated frailty severity by analyzing the G8 questionnaire, the Eastern Cooperative Oncology Group performance status, the Charlson comorbidity index, and the American Society of Anesthesiologists Physical Status System, as well as the Lee-Schonberg prognostic index. In their research, they found that the frail cohort’s complication rate was two to three times higher than that of the non-frail cohort’s. There were significant differences between the frail and non-frail groups in terms of total clinical postoperative complications, pulmonary complications, wound infections, and multiple complications, respectively [31].

In this study, we showed that increasing age is a crucial determinant for early postoperative complications, as previously demonstrated by Erekson and colleagues, who found that increased postoperative complications following gynecologic surgery were associated with dependent functional status, age ≥ 80, medical comorbidities, and accidental weight loss [32]. 

Notably, our findings suggest that incorporating frailty assessment at diagnosis or before surgery may help predict intraoperative complications in women with endometrial cancer, while BMI and age were not good predictors. We think that the crucial role of frailty as a strong predictor of adverse outcomes in our sample of older women could be attributed to impaired tissue microcirculation, which is responsible for adjustments in vascular tone to match local tissue perfusion with oxygen demand [33]. This is the pathophysiologic mechanism of different disease states that are often concomitant in frail patients, including hypertension, diabetes, and coronary artery disease [34]. Steep Trendelenburg’s position combined with pneumoperitoneum, needed in this type of surgery [35], could have contributed to impaired microcirculation of the pelvic district in frail, susceptible patients.

To the best of our knowledge, this is the first multicenter study reporting a high impact of comorbidities assessed using the mFI on intraoperative outcomes in women diagnosed with endometrial cancer. In 2020, Aloisi and colleagues assessed the characteristics of frail elderly versus non-frail elderly patients who sustained gynecologic oncology robotic surgery, finding that 8.2% of patients experienced one or more perioperative complications, of which only 4 (0.4%) were intraoperative (*p* = 0.57) [36].

Over ten years ago, Courtney-Brooks and colleagues demonstrated that assessing frailty before surgery was feasible in gynecologic oncology patients, even in a busy clinic setting, since a preoperative frailty assessment required less than 20 min in ninety-two percent of patients [27]. 

Close collaboration among gynecologists, anesthesiologists, and geriatrics could help obtain a tailored surgical approach and optimize the perioperative management of older frail women undergoing surgery for endometrial cancer in a multidisciplinary setting [37,38]. In addition, preoperative rehabilitation performed in the weeks before surgery could significantly improve functional performance, as stated by Hall and colleagues, who showed that prehabilitation could improve participants’ functional capacity after a median of five weeks of intervention before surgery [39]. 

This study has several limitations. First, it included patients with different tumor stages who underwent laparoscopic or robotic-assisted hysteroannessiectomy, even if there were no differences between frail and non-frail patients for these characteristics. Second, the mFI score was applied retrospectively to patients tracked in our prospective database based on medical comorbidities at the time of surgery, which raises the chance of reporting bias. In addition, due to this study’s design, we could not appropriately compare the frail vs. non-frail patients’ outcomes, as neither patient matching nor propensity scores could be evaluated. Moreover, using a single tool for frailty assessment may have limited value compared to the comprehensive geriatric assessment (CGA). Indeed, it has been demonstrated that the number of incorporated CGA domains greatly influences the prevalence of frailty and adequately predicts 30-day postoperative morbidity [40].

## 5. Conclusions

In conclusion, this multicentric analysis of older women with endometrial cancer who underwent robotic or laparoscopic surgery suggests that frailty, defined by an mFI ≥ 3, is associated with a significantly higher risk for intraoperative complications, supporting the practice of incorporating a frailty assessment at diagnosis or before surgery to predict the early outcome better. In our sample, frail women formed a separate clinical group among older people, confirming that frailty is not a linear extension of age and represents a challenge for care. Advanced age should also be considered an independent predictor of early postoperative complications.

## Figures and Tables

**Table 1:** Baseline characteristics of the patients (values are expressed as means ± standard deviation and numbers and percentages).

Variable	Overall (n = 577)	Non-Frail (n = 537)	Frail (n = 40)	*p* Value
**Mean Age (yrs.)**	76.45 ± 4.72	76.44 ± 4.75	76.55 ± 4.27	0.89
**BMI (Kg/m^2^)**	29.39 ± 5.92	29.35 ± 6.99	29.91 ± 4.93	0.56
**Medical Comorbidities**				
HTN (n%)	377 (65.3%)	343 (63.9%)	34 (85%)	<0.01 *
DM (n%)	130 (22.5%)	105 (19.6%)	25 (62.5%)	<0.01 *
TIA (n%)	8 (1.4%)	3 (0.6%)	5 (12.5%)	<0.01 *
CAD (n%)	18 (3.1%)	1 (0.2%)	17 (42.5%)	<0.01 *
VTE (n%)	26 (4.5%)	17 (3.2%)	9 (22.5%)	<0.01 *
Stroke (n%)	6 (1.0%)	1 (0.2%)	5 (12.5%)	<0.01 *
COPD (n%)	22 (3.8%)	16 (3%)	6 (15%)	0.01 *
Sensorial Deficits (n%)	28 (4.9%)	17 (3.2%)	11 (27.5%)	<0.01 *
Heart Failure	39 (6.8%)	19 (3.5%)	20 (50%)	<0.01 *

Abbreviations: n, number; yrs, years; BMI, body mass index; HTN, hypertension; DM, diabetes mellitus; VTE, venous thromboembolic disease; CAD, coronary artery disease; COPD, chronic obstructive pulmonary disease; TIA, transient ischemic attack; * statistically significant.

**Table 2:** Pathological characteristics of patients with endometrial cancer (values are expressed as numbers and percentages).

Variable	Overall (n = 577)	Non-Frail (n = 537)	Frail (n = 40)	*p* Value
**Histology**				0.68
Serous (n%)	38 (6.6%)	35 (6.1%)	3 (7.5%)	
Endometroid (n%)	491 (85.1%)	458 (79.4%)	33 (82.5%)	
Clear Cell (n%)	14 (2.4%)	11 (2%)	3 (7.5%)	
Carcinosarcoma (n%)	10 (1.7%)	9 (1.7%)	1 (2.5%)	
“Mixed” (n%)	8 (1.4%)	8 (1.5%)	0	
Neuroendocrine (n%)	1 (0.2%)	1 (0.2%)	0	
Adenosquamous Carcinoma	3 (0.5%)	3 (0.6%)	0	
Undifferentiated Carcinoma	4 (0.7%)	4 (0.7%)	0	
Adenocarcinoma	7 (1.2%)	7 (1.3%)	0	
Adenosarcoma	1 (0.2%)	1 (0.2%)	0	
**Grade**				0.96
IA (n%)	263 (45.6%)	246 (45.7%)	17 (42.5%)	
IB (n%)	195 (33.8%)	181(33.7%)	14 (35%)	
II (n%)	55 (9.5%)	50 (9.3%)	5 (12.5%)	
III A (n%)	11 (1.9%)	10 (1.9%)	1 (2.5%)	
III B (n%)	9 (1.55%)	8 (1.5%)	1 (2.5%)	
III C1 (n%)	23 (3.9%)	21 (3.9%)	2 (5%)	
III C2 (n%)	7 (1.2%)	7 (1.3%)	0	
IV A (n%)	3 (0.5%)	3 (0.6%)	0	
IV B (n%)	11 (1.9%)	11 (2%)	0	

Abbreviations: n: number.

**Table 3:** Complications rate in the whole sample and in frail versus non-frail patients (values are expressed as numbers and percentages).

Variable	Overall (n = 577)	Non-Frail (n = 537)	Frail (n = 40)	*p* Value
**Intraoperative Complications**				0.01 *
Yes (n%)	12 (2.1%)	9 (1.7%)	3 (7.5%)	
No (n%)	565 (98%)	528 (98.3%)	33 (82.5%)	
**Early PC**				0.06
Yes (n%)	43 (7.5%)	37 (6.9%)	17 (42.5%)	
No (n%)	534 (92.5%)	500 (93.1%)	14 (35%)	
**Delayed PC**				0.65
Yes (n%)	22 (3.8%)	21 (3.9%)	1 (2.5%)	
No (n%)	555 (96.2%)	516 (96.1%)	39 (97.5%)	

Abbreviations: n: number; PC: postoperative complications; * statistically significant.

**Table 4:** Results of multiple logistic regression analyses predicting intraoperative, early, and delayed postoperative complications. Frailty was considered a dichotomic variable (yes/no).

Variable	Odd Ratio	95% CI	SE	*p* Value
**Intraoperative Complications**				
Frailty	4.54	1.18–17.60	0.69	0.028 *
Age	1.07	0.95–1.20	0.67	0.265
BMI	1.04	0.95–1.20	0.05	0.458
**Early PC**				
Frailty	2.34	0.92–6.00	0.47	0.075
Age	1.07	1.00–1.15	0.71	0.032 *
BMI	1.04	0.98–1.09	0.35	0.201
**Delayed PC**				
Frailty	0.69	0.09–5.28	1.04	0.719
Age	0.99	0.90–1.01	0.05	0.902
BMI	0.47	0.90–1.05	0.04	0.468

Abbreviations: mFI: modified frailty index; CI: confidence interval; PC: postoperative complications; * statistically significant, SE: standard error.

## Data Availability

The data presented in this study are available upon reasonable request from the corresponding author. The data are not publicly available according to the privacy policy.
